# Supplementary material for: Hierarchical mechanism of amino acid sensing by the T-box riboswitch
Source: Nat Commun. 2018 May 14;9:1896. doi: 10.1038/s41467-018-04305-6 (PMC5951919; doi:10.1038/s41467-018-04305-6)
Supplement: Supplementary file 1 — Supplementary Information [file 41467_2018_4305_MOESM1_ESM.pdf]

Supplement to:

Hierarchical mechanism of amino acid sensing by the T-box riboswitch

Krishna C. Suddala[1,2], Javier Cabello-Villegas[2], Malgorzata Michnicka[3], Collin Marshall[2], Edward P. Nikonowicz[3], Nils G. Walter[2]

[1]Biophysics, [2]Single Molecule Analysis Group, Department of Chemistry, University of Michigan, Ann Arbor, MI 48109, USA,[3]Department of Biochemistry and Cell Biology, Rice University, Houston, TX 77005, USA. Correspondence and requests for materials should be addressed to N.G.W. (nwalter@umich.edu) and E.P.N. (edn@rice.edu).

<u>Supplementary Fig. 1, Suddala *et al.*</u>

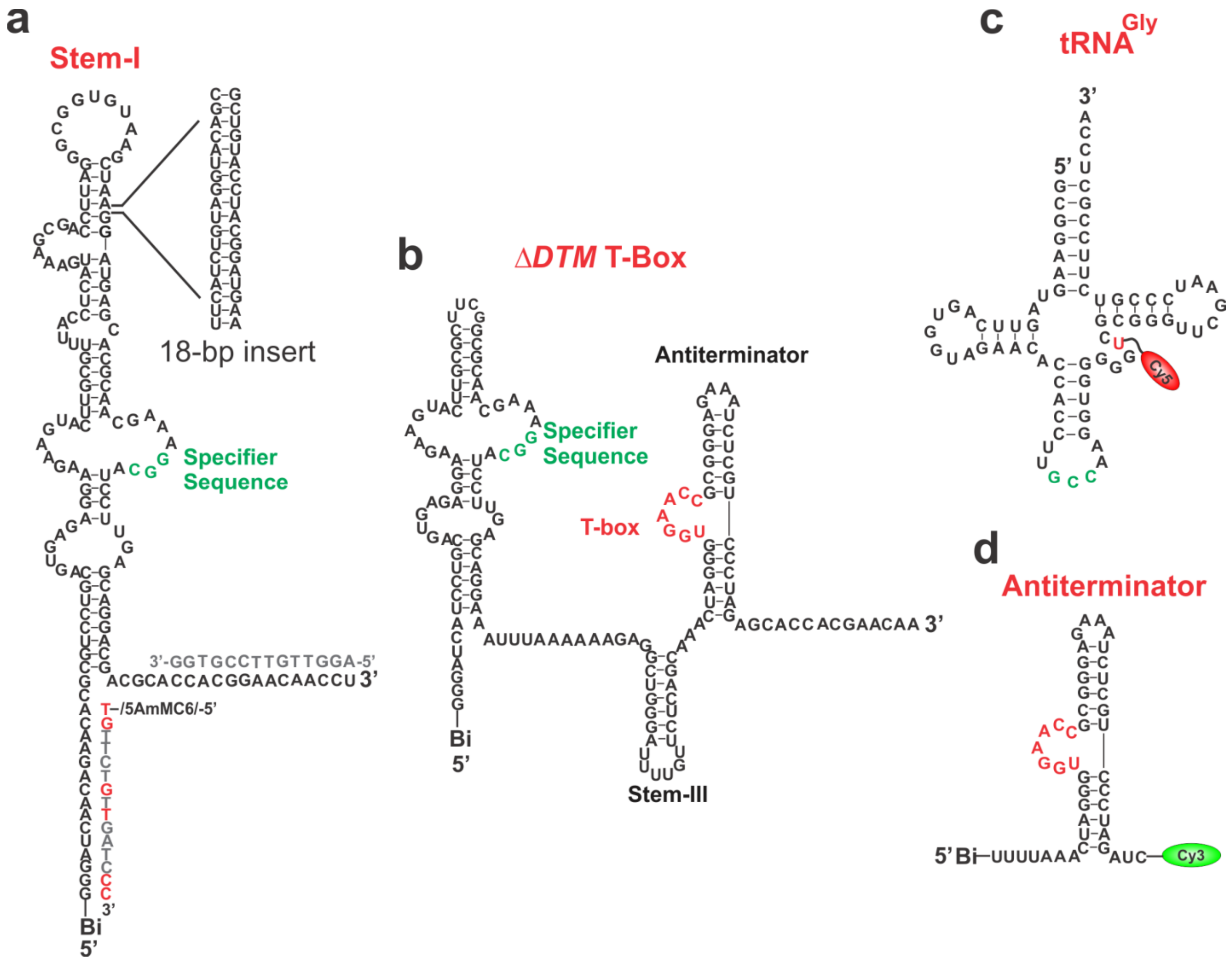

Supplementary Figure 1| Sequences and secondary structures of T-box RNA variants and tRNA^{Gly}. (**a**) Sequence and secondary structure of glyQS Stem-I construct. The sequence and location of the 18-bp insert for the Stem-I+18-bp variant is also shown, as are the sequences of the DNA and LNA oligonucleotides that are complementary to the 5' and 3'-ends of the RNA. The LNA nucleotides are shown in red. (**b**) Sequence and secondary structure of the ΔDTM T-box variant. (**c**) Sequence of tRNA^{Gly} showing the position of the Cy5 label at U46 in the variable loop. (**d**) Sequence of the isolated antiterminator T-box construct with a 3'-Cy3 label.

Supplementary Fig. 2, Suddala *et al.*

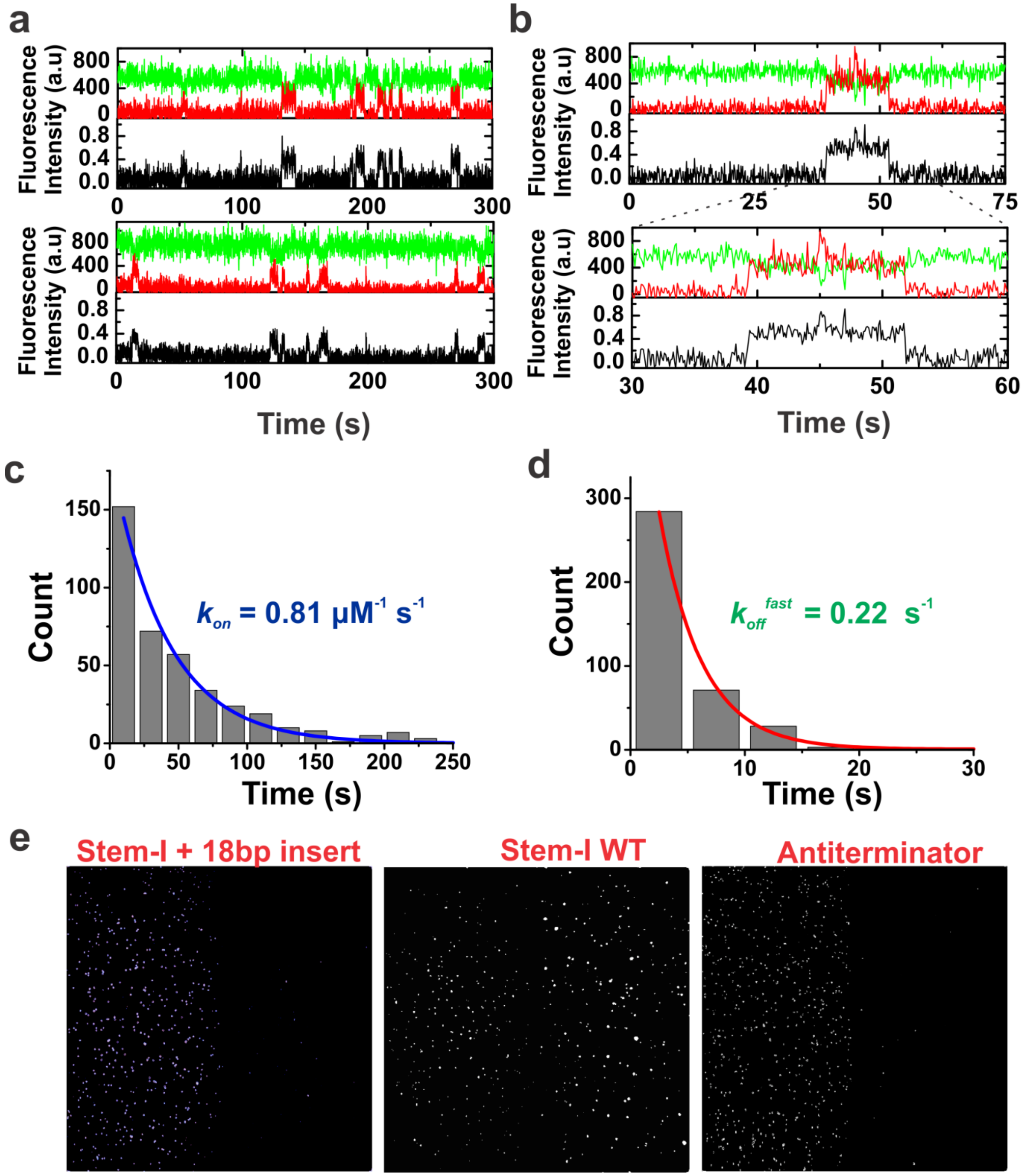

Supplementary Figure 2| tRNA binding to the isolated Stem-I fragment. (**a**) smFRET traces of tRNA binding to Stem-I showing multiple short binding events. (**b**) Example smFRET trace showing rare transitions to high (~0.8-0.9) FRET states. A closer view of the trace above is shown in the bottom panel. Dwell-time distributions for (**c**) k_{on} and (**d**) k_{off} obtained from FRET traces of tRNA binding to Stem-I. (**e**) Average image of the first 30 frames (3 s) for tRNA binding to Stem-I + 18-bp variant, WT Stem-I and isolated antiterminator variant, in the presence of 25 nM free tRNA. These images show the absence of tRNA binding for the Stem-I+18-bp and antiterminator constructs, as compared to the Stem-I WT construct.

<u>Supplementary Fig. 3, Suddala *et al.*</u>

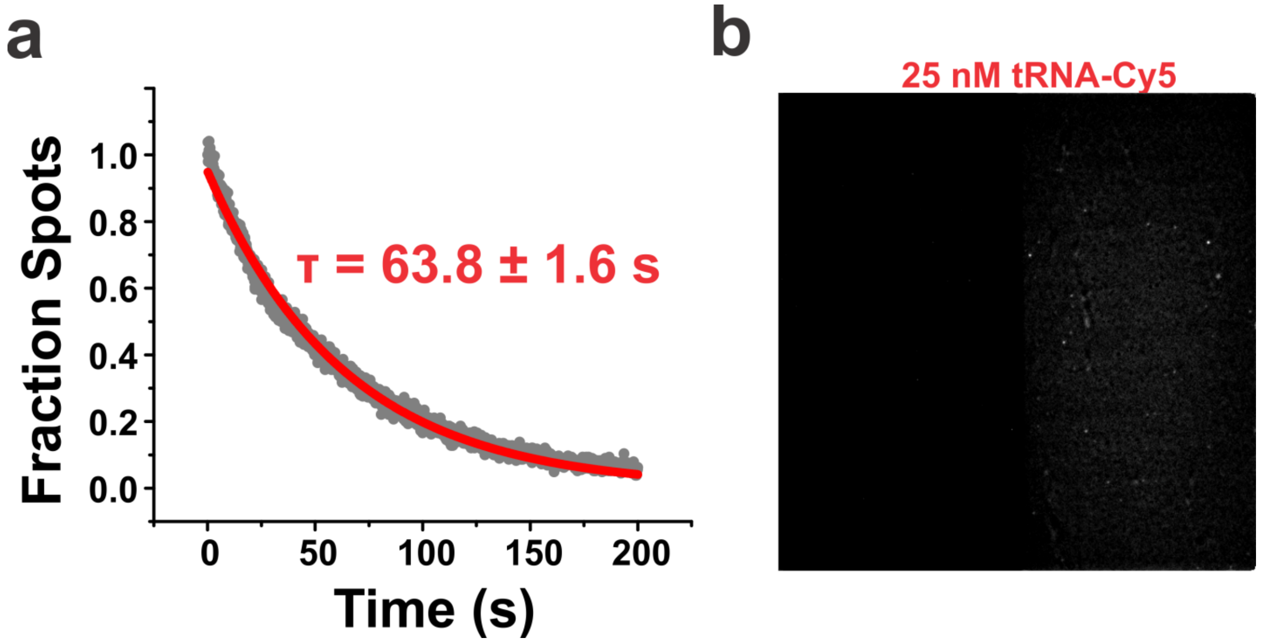

Supplementary Figure 3| Photobleaching and non-specific binding controls. (**a**) Determination of the average Cy5-photobleaching rate under our imaging conditions in standard 1x buffer with OSS. The fraction of surface bound Cy5 spots as function of time as fit with a single-exponential function, shown in red to get the photobleaching life-time. (**b**) Image showing lack of non-specific binding of Cy5 labeled tRNA to the slide surface in the absence of any immobilized T-box RNA.

<u>Supplementary Fig. 4, Suddala *et al.*</u>

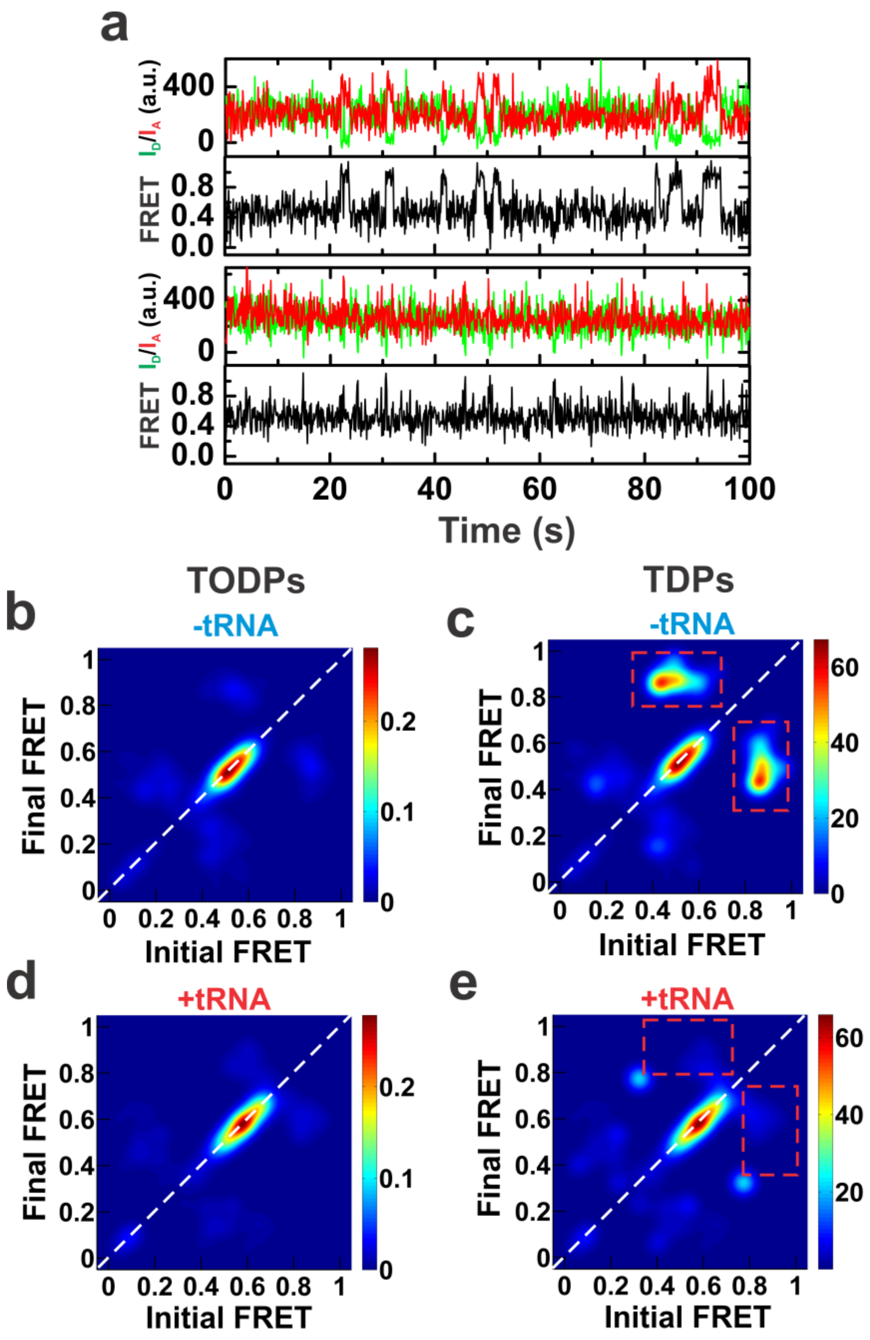

Supplementary Figure 4| Distance changes between Stem-I and antiterminator. (**a**) Additional example smFRET showing dynamic distance changes between the bases of Stem-I and antiterminator, in the absence (top) and presence (bottom) of 2 μM tRNA. Transition Occupancy Density Plots (TODPs) showing the fraction of dynamic and static traces as off- and on-diagonal contours. By contrast, Transition Density Plots (TDPs) show the number of transitions as heat maps for (**b,c**) no tRNA and (**d,e**) with tRNA. The TODPs show largely static traces as evident from the large on-diagonal contours. The TDPs show the dynamics as off-diagonal contours in the absence of tRNA, highlighted in the red box, which are clearly lost in the presence of tRNA.

Supplementary Fig. 5, Suddala *et al.*

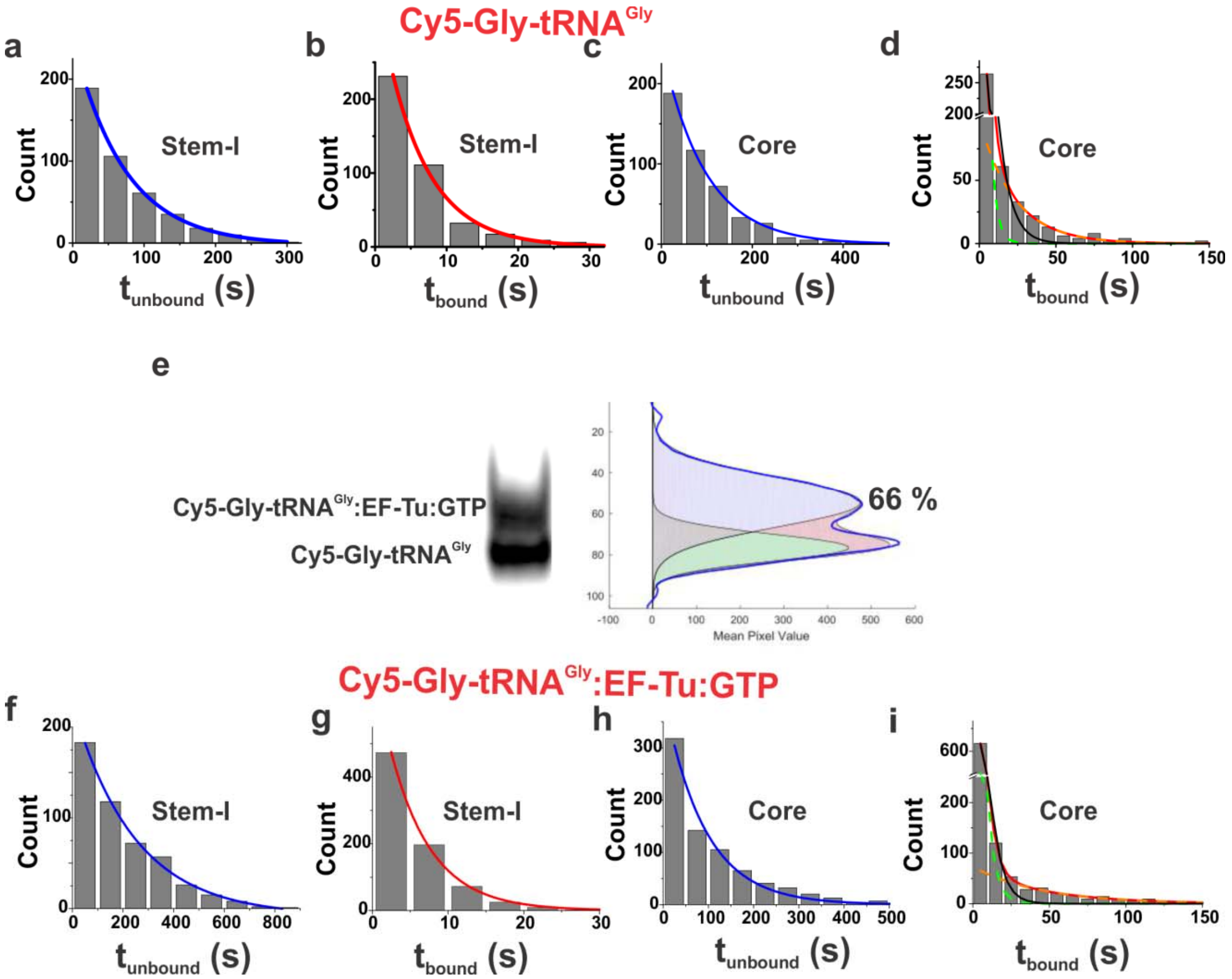

Supplementary Figure 5| Kinetics of Gly-tRNA^{Gly} and ternary complex (Gly-tRNA^{Gly} :EF-Tu:GTP) binding to Stem-I and the Core T-box riboswitch. Dwell-time distribution of $t_{unbound}$ and t_{bound} with fits to single-exponential functions shown in blue and red, respectively, for Gly-tRNA^{Gly} binding to Stem-I (**a-b**) and the core T-box riboswitch (**c-d**). The dwell-time distribution of t_{bound} in (**d**) is fit with double-exponential function (red) and the individual fits for the fast (green) and slow (orange) components are shown. The fit with a single-exponential function is shown as a black curve. (**e**) Native gel of 0.1 μM Cy5-Gly-tRNA in the presence of 2 μM EF-Tu*GTP. The upper band corresponds to the tRNA shifted by EF-Tu. Right: Quantification using GelBandFitter[1]. ~66 % of Cy5-Gly-tRNA is shifted, leading to an estimated apparent K_d of ~1.0 μM. (**f-i**) In the same order as in (**a-d**), but for Gly-tRNA^{Gly}:EF-Tu:GTP (ternary complex) binding to Stem-I (**f,g**) and the core T-box riboswitch (**h,i**). The dwell-time distribution of t_{bound} in (**i**) is fit with double-exponential function (red) and the individual fits for the fast (green) and slow (orange) components are shown. The fit with a single-exponential function is shown in black.

Supplementary Fig. 6, Suddala *et al.*

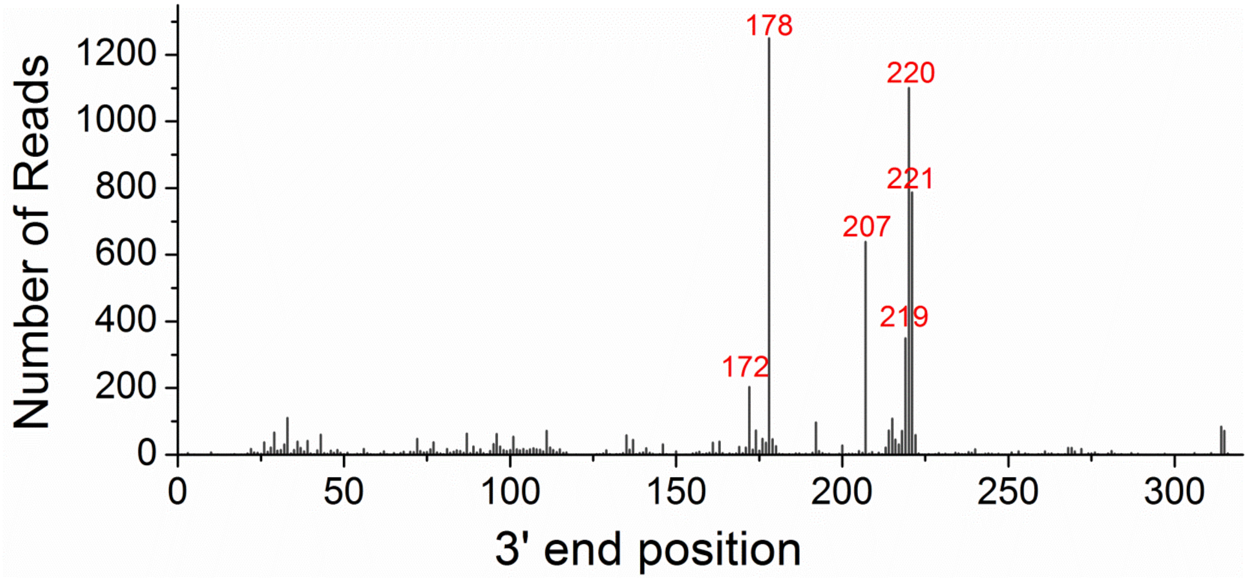

Supplementary Figure 6| NET-Seq analysis of *in vivo* RNAP pause sites in *B. subtilis glyQS* T-box riboswitch leader sequence. The number of NET-Seq reads obtained at each position in the *glyQS* leader sequence is plotted. The NET-Seq data shown is from a previous study by Larson et. al [2]. The major pause sites at positions 178 and 207 are indicated in the sequence in **Fig. 1b.**

REFERENCES

1. Mitov, M.I., Greaser, M.L. & Campbell, K.S. GelBandFitter--a computer program for analysis of closely spaced electrophoretic and immunoblotted bands. *Electrophoresis* **30**, 848-51 (2009).
2. Larson, M.H. et al. A pause sequence enriched at translation start sites drives transcription dynamics in vivo. *Science* **344**, 1042-7 (2014).
